# Making Marine Life Count: A New Baseline for Policy

**DOI:** 10.1371/journal.pbio.1000531

**Published:** 2010-10-26

**Authors:** Meryl J. Williams, Jesse Ausubel, Ian Poiner, Serge M. Garcia, D. James Baker, Malcolm R. Clark, Heather Mannix, Kristen Yarincik, Patrick N. Halpin

**Affiliations:** 1 Member, Scientific Steering Committee, Census of Marine Life, 17 Agnew Street, Aspley, Qld, 4034 Australia; 2 Alfred P. Sloan Foundation, New York, New York, United States of America; 3 Chief Executive Officer, Australian Institute of Marine Science, and Chair, Census of Marine Life Scientific Steering Committee, Townsville, Australia; 4 Member, Scientific Steering Committee, Census of Marine Life, Via Perdasdefogu, 14, 00050 Aranova, Roma, Italy; 5 Member, Scientific Steering Committee, Census of Marine Life, 8031 Seminole Avenue, Philadelphia, PA 19118, USA; 6 Principal Scientist (Deepwater Fisheries), National Institute of Water and Atmospheric Research, Wellington, New Zealand; 7 Census of Marine Life International Secretariat, The Consortium for Ocean Leadership, Washington DC, United States of America; 8 Associate Professor of Marine Geospatial Ecology, Duke University, Durham, North Carolina, United States of America

## Abstract

The Census of Marine Life aids practical work of the Convention on Biological Diversity, discovers and tracks ocean biodiversity, and supports marine environmental planning.

From the start, ocean use and resource exploitation by humans proceeded with limited knowledge of marine life and habitats. Even in the last century, biological knowledge of the oceans remained more limited than that of physical ocean processes such as storms, tsunamis from undersea earthquakes and teleconnections, like El Niño. Yet, human exploitation of the oceans is accelerating, reaching greater depths (Figure 1) and having greater impacts on marine life. Many uses interact, as when ports displace fishing, chemical industries contaminate marine life, and greenhouse gases in the atmosphere acidify and warm the oceans. Sustainable, science-based ocean policies that mitigate human impacts urgently need enhanced knowledge of marine life.

**Figure 1:**
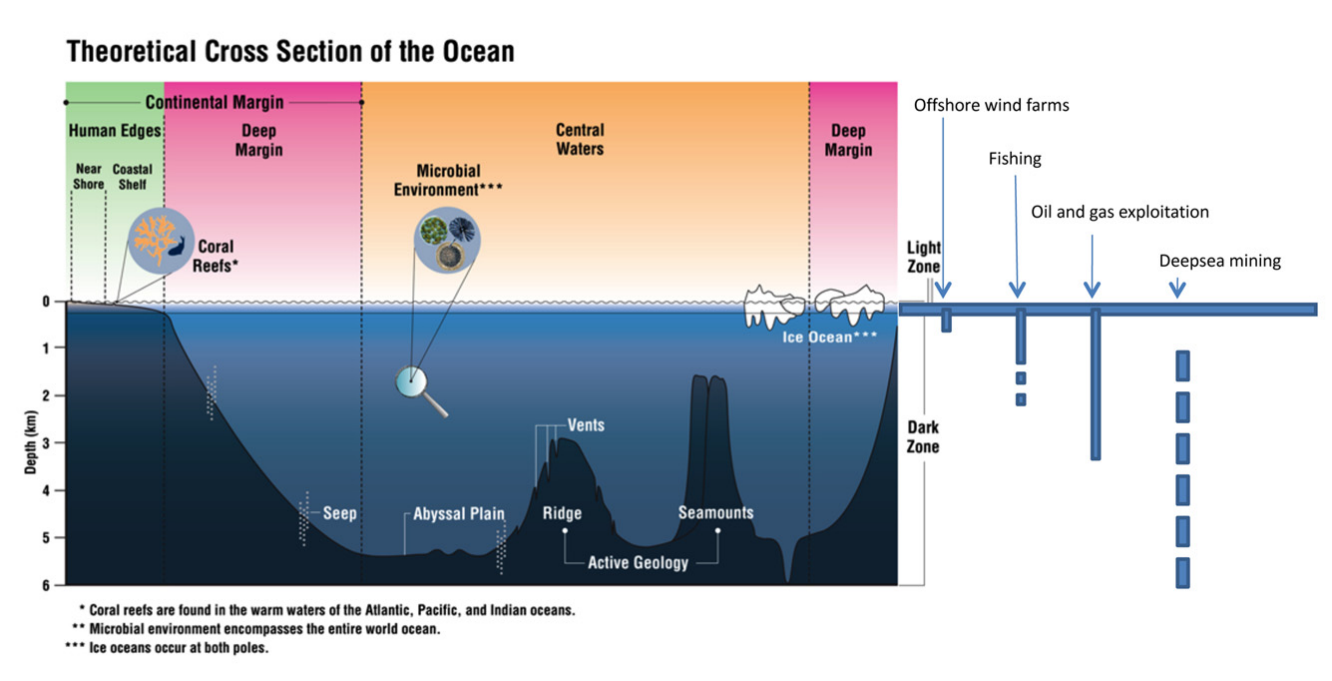
Schematic cross-section of the ocean indicating ocean realms and current (solid line) and proposed (broken line) depths of exploitation for fishing, oil and gas, deep-sea mining, and wind-farms. Wind farms: to 220m, plus offshore floating turbines anchored at greater depths (http://en.wikipedia.org/wiki/Wind_farm, accessed 25 May 2010). Fishing: current commercial fishing occurs between 1000 to 1400m; fishing deeper than 1500m is not constrained by technical limitations and vessels could modify equipment to suit. (F. Chopin, FAO, personal communication). Oil and gas: 3,000m (*The Economist*, March 4 2010). Deep-sea mining: 1,000–6,000m (*Technical Study No. 2*, International Seabed Authority 2002). Image: CoML and Meryl Williams.

## The Origin and Work of the Census of Marine Life

**Figure 2:**
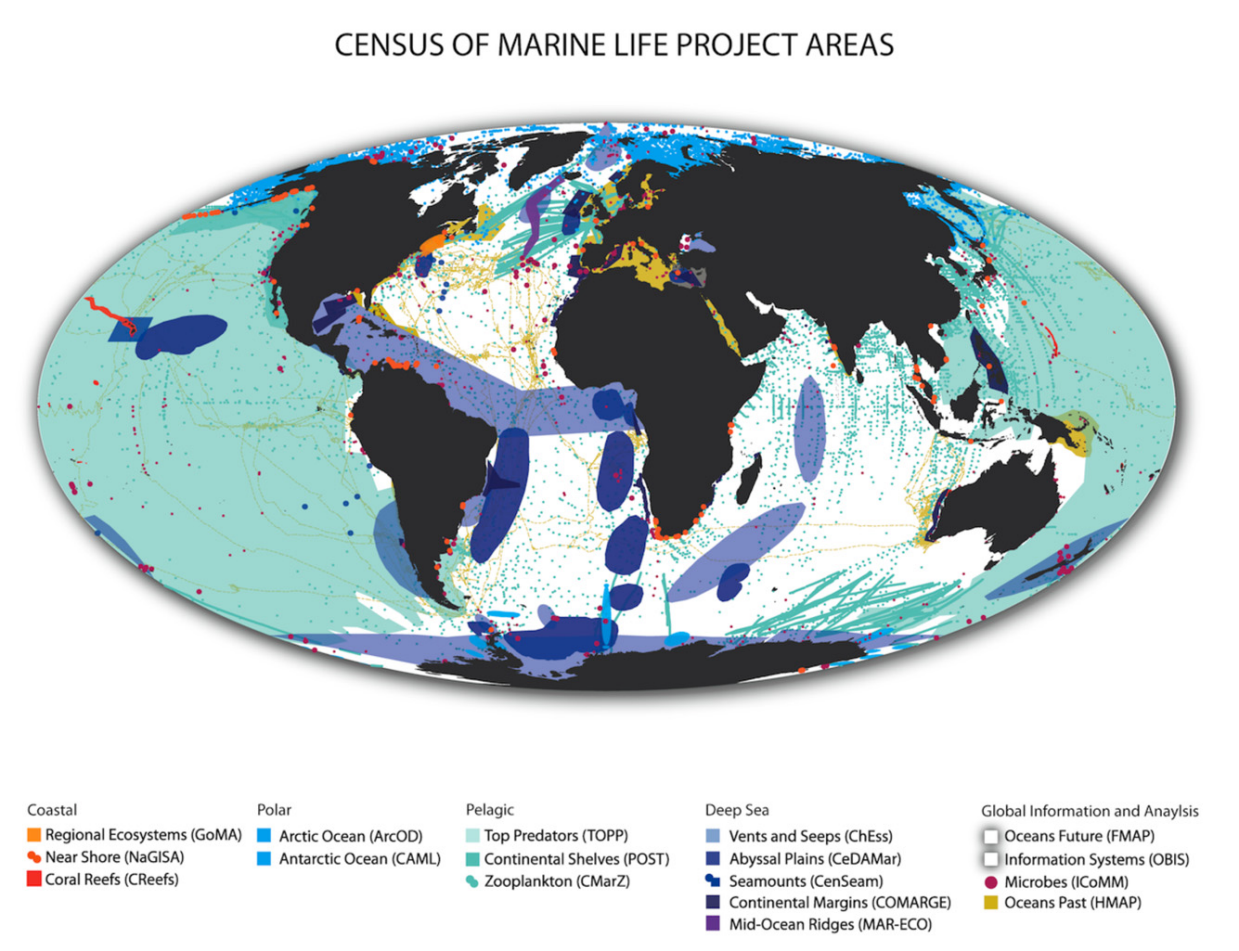
Census of Marine Life project areas. Image: CoML.

Launched in 2000, the decade-long Census of Marine Life partnership (CoML or the Census - http://coml.org) converged with advances in information, communication, genetic, sensory, and acoustic technologies to spur knowledge of marine life. It sought to expand the known, shrink the unknown and set aside the unknowable. The Census received core funding and intellectual guidance from the Alfred P. Sloan Foundation. Its strategic goal was to comprehend the diversity, distribution and abundance of marine life, from microbes to whales. The Census spanned all ocean realms, from coast to abyss, from the North Pole to Antarctic shores, from the long past to the future (Figure 2). It systematically compiled information from new discoveries and historic archives and made it freely accessible. It employed conventional research ships and sampling, divers and submersible vehicles, genetic identification, electronic and acoustic tagging, listening posts and communicating satellites [1].

**Figure 3:**
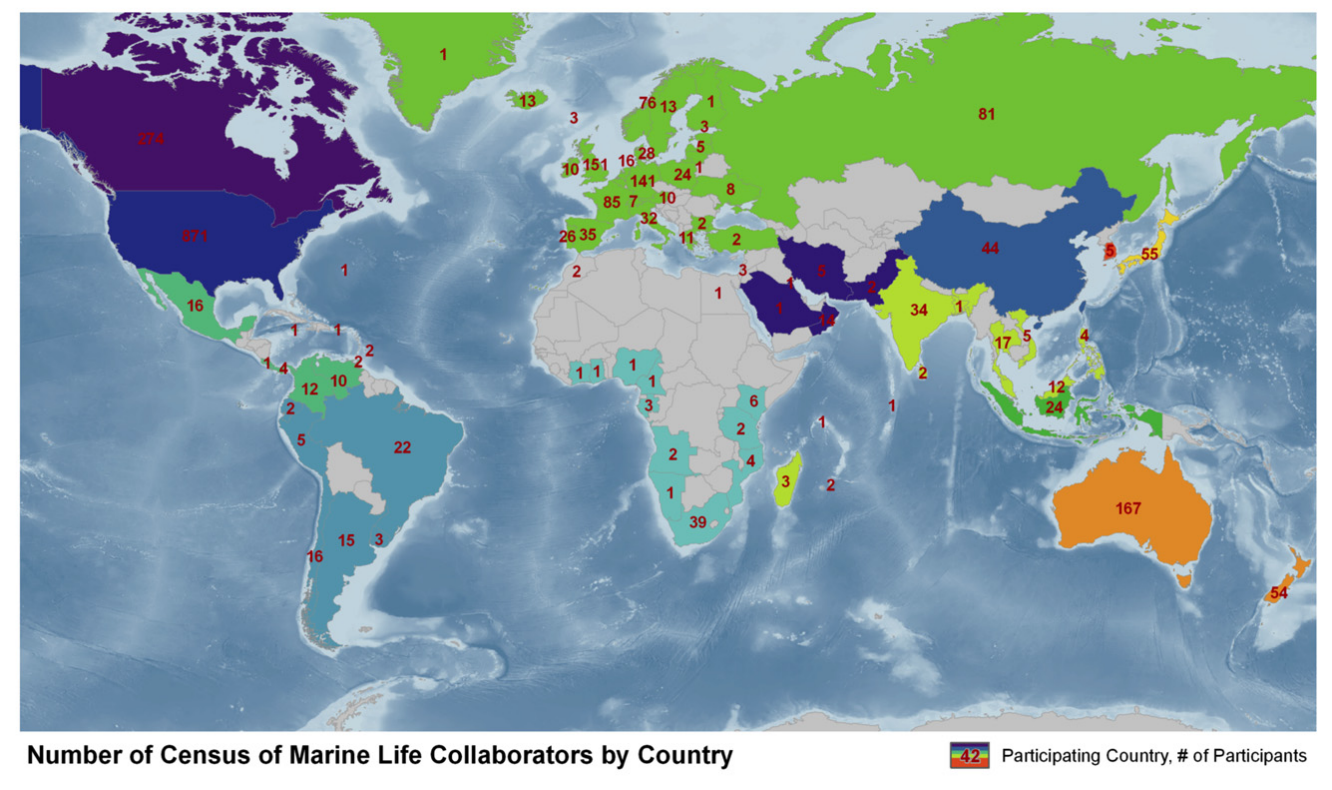
Participation by country and region. Countries coded with the same color collaborate in a regional implementation committee and numbers within country borders indicate the number of collaborating Census scientists for that country. Image: CoML

More than 2,700 scientists from more than 80 nations and 540 scientific expeditions using $650M (est.) from nearly 500 sources of funding and in-kind contributions mobilized around 17 Census and five affiliated projects, each headed by leading scientists. Census governance balanced strategy and coordination with project management that gave experts the freedom to innovate and ensured global reach. The Census, through its international oversight bodies, projects, and 13 National and Regional Implementation Committees spanning the globe (Figure 3), has already contributed 2,600 papers to the scientific literature, many in special editions of specialist journals.

The Census partnership produced results on a scale never before achieved for marine life and created a new baseline of knowledge. From Census specimens, more than a thousand new species, several new genera and a new family have already been named and more than 5,000 new candidates have been collected and are waiting to be named [2]–[4]. Using acoustic technologies, Census scientists discovered a shoal of herring as large as Manhattan off the coast of New Jersey [5] and tracked Pacific salmon from their natal rivers to Alaska [6]. Amidst the new discoveries, however, are sobering insights into historical depletions. From historic records, the Census showed that people have depleted populations of marine species worldwide over hundreds and sometimes thousands of years, changing the structure of marine-life communities, the profitability of harvesting and the ability to recover [7]. Emerging discoveries on the diversity and distribution of microbes, the largest source of marine biomass [8], will be central to tracking the impacts of more acidic, warmer, low oxygen oceans under climate change.

The Census is bequeathing such legacies as the Ocean Biogeographic Information System (OBIS – http://iOBIS.org ), which is now incorporated into UNESCO's International Oceanographic Commission as part of the International Oceanographic Data and Information Exchange (IODE). The Census stimulated ongoing partner projects including the Encyclopedia of Life (a webpage for every species), the Barcode of Life (short DNA identifiers for every species), and the Ocean Tracking Network (observations of animal movements spanning the globe). Some Census field projects will continue in different forms. For example, two animal tracking projects have joined forces and provided prototype technology for the Ocean Tracking Network; the six deep-sea projects have collaborated on the Synthesis of the Deep-sea projects of the Census of Marine Life (SYNDEEP); and the Gulf of Maine Area Program has borne an offspring called Canada's Healthy Ocean Network. The History of Marine Animal Populations has spawned a new field of study that integrates scholars in social and natural sciences and humanities, and the work of the Future of Marine Animal Populations will continue through a team at Dalhousie University. Another continuing collaboration is the Global Ocean Biodiversity Initiative (GOBI – http://www.gobi.org), which involves the International Union for Conservation of Nature (IUCN), the German government, several United Nations and non-government agencies, and many Census projects that are identifying places in the open oceans and deep sea deserving protection.

Successful policy acceptance and adoption requires a solid foundation of public awareness. To achieve this, Census discoveries were brought to public notice. The Census made extensive use of new media so that, for example, millions of people watched “great turtle races” tracking turtle migrations on live TV. Aided by press releases, Census discoveries have earned global media attention. The Census cooperated with the cutting edge team of Galátee, Inc., led by Jacques Perrin and Jacques Clouzaud, to produce the film *Oceans*, which premiered in 2010 and is already one of the highest grossing documentaries ever.

What was unpredicted at the start of the Census was the depth of policy interest in the results. Already, the Census results have started to influence policies and management in such bodies as the International Seabed Authority. Three examples of the uses of Census expertise are: (1) assisting the Convention on Biological Diversity (CBD) as it defines potential protected areas in the open ocean and deep seas, (2) supporting marine planning for regions and ecosystems, and (3) contributing marine biology observations for the Global Earth Observing System of Systems (GEOSS) of the intergovernmental Group on Earth Observations (GEO).

## Convention on Biological Diversity Addresses the Open Oceans

The Census' discovery, mapping and counting of species measures biodiversity. The international legally binding treaty on biodiversity is the Convention on Biological Diversity (CBD) adopted in Rio de Janeiro in June 1992. A decade later in 2002, the World Summit on Sustainable Development (WSSD) agreed upon 2012 as the target year to establish an international network of representative marine protected areas [9].

The CBD enshrined national sovereignty over biodiversity, but this left marine life in the 64% of the oceans outside national jurisdictions largely unprotected. Several regional fisheries management organizations and regional coastal and ocean management agencies have been established in recent decades and are working towards regulating use of shared species and ocean regions, including areas of the open ocean and deep seas. However, marine biodiversity protection is only lately entering the considerations of most of these bodies, often with reference to WSSD [9]. The CBD is also redressing this neglect of biodiversity outside national waters and has established scientific criteria for “ecologically and biologically significant areas” (EBSA) [10]. The EBSA scientific criteria are: (1) uniqueness or rarity; (2) special importance for life history of species; (3) importance for threatened, endangered, or declining species and/or habitats; (4) vulnerability, fragility, sensitivity, and slow recovery; (5) biological productivity; (6) biological diversity; and (7) naturalness. The EBSA criteria were then tested by pilot illustrations for 15 different areas/species.

Here is where CoML comes in. In collaboration with the Global Ocean Biodiversity Initiative, Census researchers contributed several critical pilot illustrations from OBIS and Census-led field and service projects: CenSeam (seamounts), MAR-ECO (Mid-Atlantic Ridge), TOPP (Tagging of Pacific Predators), OBIS, and the Mapping and Visualization (M&V) project.

**Figure 4:**
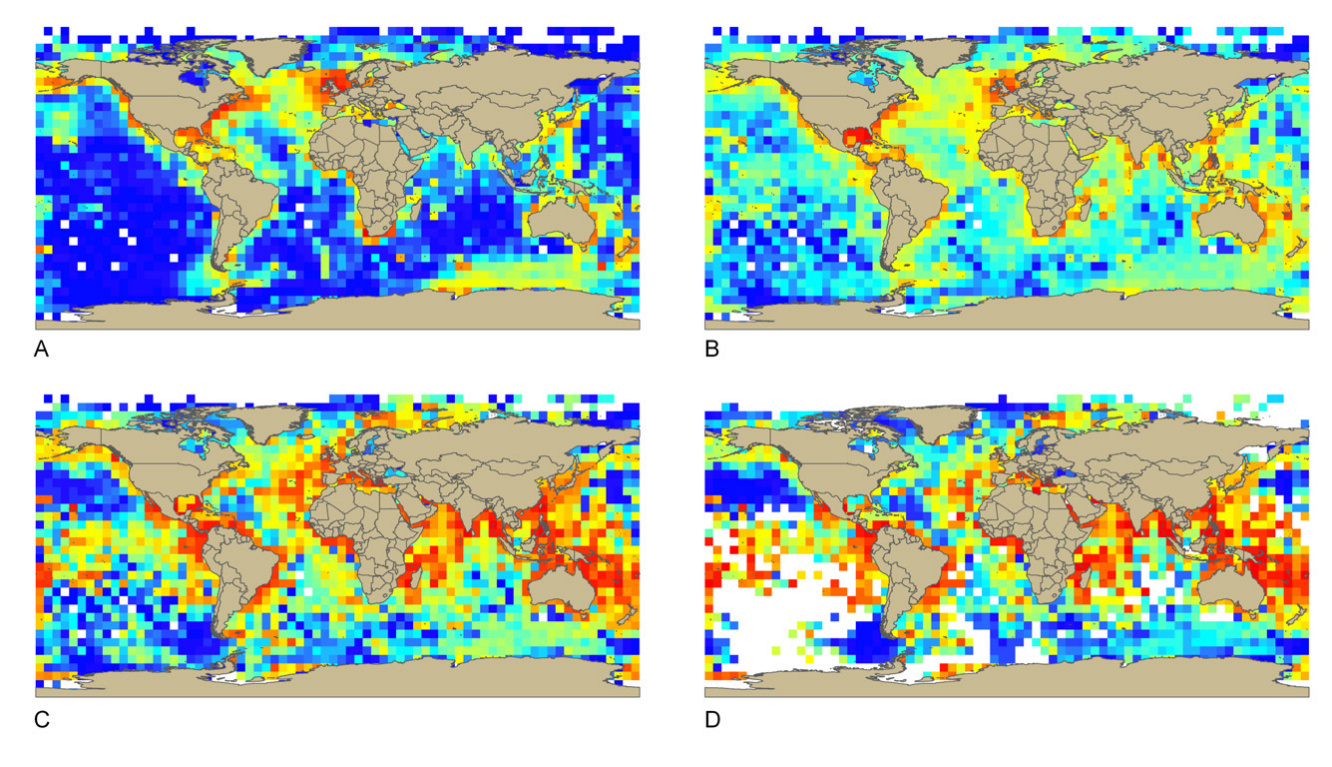
Four maps used for Convention on Biological Diversity Ecologically and Biologically Significant Areas Criterion 6, Biological diversity (Annex of reference 11). (a) total records in OBIS, corrected for differences in surface area between squares on different latitude; (b) the total number of species, corrected for differences in surface area between squares on different latitude; (c) Shannon Index; (d) Hurlbert's Index, *es(50)*.

This pilot exercise demonstrated the importance of organized publically accessible data portals such as OBIS that were able to deliver up the results of over 800 existing, quality controlled data collections, including all the data gathered by Census projects. For example, CBD's Criterion 6 concerning biological diversity defines an EBSA as an area containing relatively more diversity of ecosystems, habitats, communities, or species, or an area with more genetic diversity. To investigate global scale patterns, Census scientists provided the CBD with analysis of the more than 22 million records then in OBIS. They estimated several biodiversity indices corrected for intensity of sampling and for broad global patterns of marine biodiversity already known (Figure 4). EBSA Criterion 7 (naturalness) used the example of the southeast Atlantic seamounts. This illustration combined inputs from Census projects, such as seamount and historical trawl fishing locations from CenSeam, and biological sampling from OBIS/Seamounts Online, with human impact compilations [11],[12].

Input from Census researchers was also important in FAO discussions on management of deep-sea fisheries on the high seas, providing background information to national delegates formulating the final set of international guidelines [13],[14].

## Planning for Regions and Ecosystems

Akin to land and urban planning, marine planning has arisen to provide order and predictability to the multiple ocean uses at scales smaller than those of the global conventions such as the United Nations Convention on the Law of the Sea and the CBD. The ecosystem and precautionary approaches to planning and management have developed to encompass conservation objectives. These approaches are enshrined in recent global instruments, especially the 1995 United Nations United Nations Agreement for the Conservation and Management of Straddling Fish Stocks and Highly Migratory Fish Stocks (United Nations Fish Stocks Agreement), wherein article 5f is binding on signatories to maintain biodiversity, and the 2002 Plan of Implementation of the World Summit on Sustainable Development.

Plans for multiple uses and with multiple objectives are displacing simple plans for single uses and objectives, e.g., plans for conserving ecosystems like coral reefs, seamounts, regions like Australia's Great Barrier Reef, the Mediterranean and Baltic Seas, and the United States of America's ocean coasts and Great Lakes have become more common [15]. Ecosystem approaches and marine spatial planning both require useable knowledge of marine-life diversity, distribution, and abundance, coherent across environment and industry decision-making frameworks [16]. The Census approach emphasized validated, geographically and time-referenced biological data, and technologies that capture the dynamics of individual organisms and animal populations throughout seasons and life cycles and through history.

For example, data from Census projects CeDaMar (abyssal plains) and CenSeam (seamounts) fed into designing a “Preservation Reference Area” network in the Clarion-Clipperton Fracture Zone of the central Pacific Ocean by the International Seabed Authority to manage potential mining for polymetallic nodules [17]. Through modeling, Census scientists have predicted the likely distribution of deep-sea corals that are indicator species and highly vulnerable to impacts from fishing or mining [18]. Regional fisheries management organizations, such as the South Pacific Regional Fisheries Management Organization, have used indicator species to predict where habitats sensitive to fishing might occur in data poor regions [19].

Census researchers played a major role in the development of the UNESCO Global Open Oceans and Deep Seabed (GOODS) biogeographic classification. The classification is designed to identify where industrial uses of the ocean are incompatible with biodiversity conservation and to protect representative marine life and ecosystems and thus aids marine planning [20].

## International Ocean Observation Systems

The intergovernmental Group on Earth Observations (GEO) is coordinating efforts to build a Global Earth Observation System of Systems (GEOSS). In 2008, GEO established a Biodiversity Observation Network (GEO-BON) as one of nine Societal Benefits Areas (http://www.earthobservations.org/geoss_bi.shtml) [21]. Effective and efficient observation of more than 200,000 species of marine animals and perhaps tens of millions of types of marine microbes present great scientific and technological challenges. Existing long-time series of marine life are rare and narrow in scope, such as the Continuous Plankton Recorder in the North Sea and North Atlantic (Sir Alistair Hardy Foundation for Ocean Science, http://www.sahfos.ac.uk/sahfos-home.aspx, since 1931), long-term fisheries surveys for North Sea groundfish (the International Bottom Trawl Survey (http://www.ices.dk/datacentre/datras/survey.asp, since 1960), the United States of America (since 1963) [22], and intermittent surveys from the 1920s in Asia [23]. The paucity of biological time series contrasts with the more numerous marine chemical and physical data series captured by remote sensing and such tools as drifting buoys and active float systems.

By making the oceans more “transparent” and accessible, new technologies such as demonstrated by the Census are relieving this deficiency for biology [1],[24]. For example, individual Pacific salmon (*Oncorhynchus* spp) were tracked over thousands of kilometers using tags that emit individually coded acoustic pulses to coastal receivers [6]. Via tags, how marine mammals use major oceanic features such as frontal zones under ice has been mapped [25]; new rapid genomic techniques and databases (e.g., DNA barcoding, 454-pyrotag sequencing [26] and MICROBIS – http://icomm.mbl.edu/microbis/) are rewriting knowledge of marine biodiversity and marine-life abundance. The CReefs project of the Census developed a new automated structure, (Autonomous Reef Monitoring Structures (ARMS)), 500 of which are now deployed in the Pacific and Indian oceans and the Caribbean, collecting specimens and ecological data to monitor tropical coral reef biodiversity [27].

Notwithstanding the urgency to monitor marine life, scientists and policy makers have yet to implement a set of core observing systems for a comprehensive “Bio-GOOS” [28]. The outputs from the Census will be a valuable input to such a comprehensive system.

## Reflections

With the wisdom of hindsight, what could the Census have done differently for greater policy impact? Two aspects come to mind: the possible effects of earlier policy engagement and earlier globalization.

The Census engaged with end-users relatively late in the decade. As the Census was primarily a discovery program and was not policy-directed, we were surprised at the demand for the Census to help inform policy. The demand partly derived from international commitments such as the growing list of CBD provisions, the 2002 WSSD and national laws that now oblige maritime countries to assess the status and outlook for marine life in their waters and oceans beyond. The other drivers for Census-type information were increased evidence of impacts and raised public awareness. Broader partnerships with bodies outside scientific research agencies are vital in science-policy engagement. For example, the Census partnership with IUCN has been successful on several levels, as has the Memorandum of Cooperation the CBD. These complementary partnerships enabled the Census to stay focused on unbiased science while still being able to link into the policy sphere.

Possibly, broadening the delivery model beyond scientific publications and public outreach could have had earlier impact. For example, Census scientists who engaged in delivering policy-relevant advice on high seas and seamounts fisheries [18] learned the importance of thinking outside their national objectives. They had to look at the bigger picture and access other ideas, other data, and the demands of other than their home countries. To arrive at robust advice, they had to consider generic drivers of ecosystem change on seamounts and more international and global management issues. Further, having started late in deriving the policy relevance of Census results, scientists have had to be creative to explain post hoc the usefulness in policy-relevant terms. However, neither the Census nor other bodies could have readily agreed program policy targets in advance without risking too much dispersion and losing sight of the essential science vision of the Census. Perhaps a breadth of vision in collecting basic knowledge is essential in meeting the future needs of marine management and policy?

The second aspect was underestimating the challenge of moving from expeditionary science focused on global questions delivered by scientists from established institutes to a global initiative that involved scientists from many coastal countries. National and regional scientists will have long-term carriage of policy advice to decision makers. Capacity building was not an explicit objective of the Census and yet a great deal of capacity was built. However, more focus on NRICs, and/or more NRICs, could have led to more lasting policy impacts from the Census.

With these reflections on possible improvements and the overall achievements of the Census, we conclude that investing in scientific knowledge of marine life, new discovery, and monitoring technologies and extensive databases within and across ocean use and conservation helps meet the growing demand for better ocean policies. Indeed, a significant opportunity remains to continue this work in an international and cooperative manner post the first 10 years of the Census.
